# *In Vitro* and *In Vivo* Evaluation of a Hydrogel Reservoir as a Continuous Drug Delivery System for Inner Ear Treatment

**DOI:** 10.1371/journal.pone.0104564

**Published:** 2014-08-08

**Authors:** Mareike Hütten, Anandhan Dhanasingh, Roland Hessler, Timo Stöver, Karl-Heinz Esser, Martin Möller, Thomas Lenarz, Claude Jolly, Jürgen Groll, Verena Scheper

**Affiliations:** 1 Department of Otolaryngology, Hannover School of Medicine, Hannover, Germany; 2 University of Veterinary Medicine Hannover, Foundation, Institute of Zoology, Hannover, Germany; 3 MED-EL Innsbruck, Research & Development, Innsbruck, Österreich; 4 Interactive Materials Research–DWI e.V. and Institute of Technical and Macromolecular Chemistry, RWTH Aachen University, Aachen, Germany; 5 J.W. Goethe University Hospital and Faculty of Medicine, Department of Otolaryngology, Frankfurt am Main, Germany; 6 University of Würzburg, Department of Functional Materials in Medicine and Dentistry, Würzburg, Germany; 7 Institute of Audioneurotechnology, Hannover School of Medicine, Hannover, Germany; University of South Florida, United States of America

## Abstract

Fibrous tissue growth and loss of residual hearing after cochlear implantation can be reduced by application of the glucocorticoid dexamethasone-21-phosphate-disodium-salt (DEX). To date, sustained delivery of this agent to the cochlea using a number of pharmaceutical technologies has not been entirely successful. In this study we examine a novel way of continuous local drug application into the inner ear using a refillable hydrogel functionalized silicone reservoir. A PEG-based hydrogel made of reactive NCO-sP(EO-*stat*-PO) prepolymers was evaluated as a drug conveying and delivery system *in vitro* and *in vivo*. Encapsulating the free form hydrogel into a silicone tube with a small opening for the drug diffusion resulted in delayed drug release but unaffected diffusion of DEX through the gel compared to the free form hydrogel. Additionally, controlled DEX release over several weeks could be demonstrated using the hydrogel filled reservoir. Using a guinea-pig cochlear trauma model the reservoir delivery of DEX significantly protected residual hearing and reduced fibrosis. As well as being used as a device in its own right or in combination with cochlear implants, the hydrogel-filled reservoir represents a new drug delivery system that feasibly could be replenished with therapeutic agents to provide sustained treatment of the inner ear.

## Introduction

Hearing loss affects approximately 278 million people worldwide. Next to infectious causes like meningitis, measles, mumps and chronic ear infections, hearing impairment is commonly triggered by exposure to excessive noise, head and ear injury, ageing and the use of ototoxic drugs [1]. Since sensory cells of the inner ear develop exclusively during embryogenesis and are not programmed to regenerate postnatally in mammals [2], in many cases hearing ability can only be regained by the insertion of a cochlear implant (CI) [3].

After CI surgery the acuity of residual hearing and that of CI-mediated hearing are often affected by postoperative intra-cochlear fibroblast growth and a delayed degeneration of neuronal tissue. A histological evaluation of human temporal bones from cochlear implant patients showed fibrous growth formation in 57% of examined cases. The fibrous growth formation were believed to be as a result of intra-cochlear mechanical trauma to the fine structures caused by electrode insertion as well as a foreign body reaction of the host tissue to the implant [4]. As a consequence, increased impedance, reduced speech perception and functional derogation of the device itself may take place [5].

Locally applied glucocorticoid receptor agonists like dexamethasone suppress inflammation in the inner ear and consequently prohibit growth of fibrosis related connective tissue expanse, cell degeneration and loss of residual hearing [6], [7]. Due to the relative difficulty in accessing the inner ear, the maintenance of sufficient therapeutic levels of this agent over a prolonged period has been problematic. Available application aids like pump systems and intra-cochlear electrode array loaded with dexamethasone have limited delivery duration, are not rechargeable and/or have to be detached after entirely emitting the drug (reviewed by [8]). Polyethylene glycol (PEG) based hydrogels are one of the widely studied biomaterial as tissue culture scaffold and as drug loading and delivery system [9]–[11]. Briefly, hydrogels are three dimensionally cross-linked hydrophilic polymer chains that exhibit strong swelling in water and available as degradable or non-degradable depending on the polymer chain chemistry [12]. Non-degradable hydrogel in the free form state loaded with any water soluble low molecular weight drug molecules will be diffused out completely within 24–48 hours depending on how big is the volume of release medium. For the delayed release of drug for at least 4 week time for inner ear treatment, the drug loaded hydrogel has to be physically restricted from release medium which can be achieved by incorporating the drug loaded hydrogel within the thin electrode array. The electrical impedance of the CI which is a hint of fibrous tissue growth is largely taking place in the first 4 weeks of implantation [13].

We have previously shown that six-arm star shaped poly-(ethylene oxide-*stat*-propylene oxide) prepolymers with 80% ethylene oxide (EO) content in the backbone and reactive isocyanate groups at the distal ends of the arms (NCO-sP(EO-stat-PO) can be used to prepare ultra-thin biocompatible coatings that very efficiently minimize unspecific protein adsorption [14]. We have also shown that NCO-sP(EO-stat-PO) may be used to prepare physiologically stable three-dimensional hydrogels [15] and as cross-linker to polysaccharides [16].

In this study, we evaluated the physiologically stable NCO-sP(EO-stat-PO) hydrogel as drug carrier of dexamethasone 21-phosphate disodium salt (referred to as DEX in the manuscript), a hydrophilic modification of dexamethasone with high water solubility, in silicone tubes that connect the inner ear with an external potentially refillable drug reservoir. In order to evaluate the potential use of this hydrogel system together with an implant, we have assessed the processes regarding its filling into silicone tubes, drying for shelf-storage, heat-treatment for sterilization and re-swelling after time to examine tight fit at the inner lumen of the silicone tube. Drug release from the hydrogels was first examined as free gels without outer steric restriction in order to show pure diffusion controlled release. Subsequently, drug release kinetics were examined with the hydrogel filled into a silicone tube with one end open and PBS (pH 7.4) as the release medium. Additionally, the hydrogel was studied as drug diffusion gateway for a drug reservoir made of silicone tubing. In order to understand the drug diffusion behaviour, the hydrogel was primed and replenished with DEX. For *in vivo* evaluation, hydrogel reservoirs were used to deliver DEX into the inner ear in a guinea pig model. Reservoirs primed with PBS only as well as reservoirs being implanted and immediately explanted served as control. The response of the cochlea in terms of foreign body reaction and hearing loss were examined four weeks following the implantation procedure.

## Materials and Methods

### 1. *In vitro* experiments

Star-shaped poly-ethers with a backbone of statistically copolymerized 80% ethylene oxide and 20% propylene oxide, molecular weight of 18000 g/mol (PD = 1.15) with isocyanate (NCO) end groups (NCO-sP(EO-stat-PO) was synthesized as described previously [17]. An overview on the chemical structure of the gel precursor and the cross-linking reaction is shown in Fig. S2. Dexamethasone 21-phosphate disodium salt (DEX; molecular weight 516.4 g/mol) was purchased from Sigma-Aldrich Germany. Silicone tubes of 6 cm length with an internal diameter of 0.31 mm and an outer diameter of 0.64 mm were kindly provided by MED-EL GmbH, Austria.

#### 1.1. Hydrogel preparation and determination of the swelling ratio

Hydrogels were prepared by dissolution of the reactive NCO-sP(EO-*stat*-PO) precursors in water as described before [15]. The cross-linked hydrogels were dried to xerogel condition in triplets in a vacuum chamber overnight and the dry weights were measured. Xerogels were then placed in an excess of phosphate buffered saline (PBS; pH 7.4) at 37°C for studying its swelling kinetics. The weight of the swollen gel was measured at specific time intervals until it reached its equilibrium swelling state. The swelling ratio is determined by the ratio of dry weight of the gel (W_d_) to the swollen weight of the gel (W_s_): swelling ratio  =  (W_d_/W_s_) ×100.

#### 1.2. Drug release studies from free 3D hydrogels

NCO-sP(EO-stat-PO) was dissolved in a 50 mg/mL (w/v) DEX solution in PBS (pH 7.4) to a final concentration of 20% (w/v). Subsequently, 0.5 mL of this solution (containing 25 mg DEX) was placed into a Teflon mould of 15 mm diameter and 3 mm depth and covered airtight for 24 hours. After completed cross-linking, the DEX-loaded hydrogel was dried to zerogel in a vacuum chamber and then placed in PBS at 37°C for swelling and release of the drug molecules. The amount of drug released in to the PBS was measured by CARY 100 Bio UV-visible spectrophotometer at selected time intervals until the minimum detection limits were reached. The wavelength used to detect DEX was 238–242 nm.

#### 1.3. Drug release from hydrogels in silicone tubes

PBS solution containing DEX (50 mg/mL) was used to dissolve homogenously the hydrogel precursor to obtain a 20% w/v concentration. By use of an insulin syringe avoiding air bubbles, the gellifying solution was sucked inside the silicon tubes of length 60 mm and an inner diameter of 0.31 mm with one end open and the other end closed. The silicone tubes were sealed for 24 hours to allow complete gelation and to prevent drying. Subsequently the silicone tubes containing the hydrogel were placed in PBS at 37°C to examine the release of DEX as mentioned in section 1.1.2.

#### 1.4. Hydrogel as diffusion gate in drug reservoir

NCO-sP(EO-*stat*-PO) was dissolved in PBS and sucked inside a silicone tube from the open end for a length of 10, 5 and 2 mm and maintained undisturbed for 24 hours. After cross-linking of the hydrogel, the remaining length of the silicone tube between the closed end and the hydrogel was filled with DEX solution (100 mg/mL) using an insulin syringe. This set-up was again placed in PBS at 37°C to investigate the release profile of DEX as mentioned in section 1.1.2.

### 2. *In vivo* experiments

Normal hearing Dunkin Hartley guinea pigs (n = 28; Harlan-Winkelmann GmbH, Borchen, Germany) of both sexes, weighing about 400 g were used. All experiments were carried out in accordance with the institutional guidelines for animal welfare of Hannover Medical School following the standards described by both German laws on protecting animals (Tierschutzgesetz) and the European Communities Council Directive 86/609/EEC for the protection of animals used for experimental purposes. The experiments of this study were approved by the regional government (Niedersächsisches Landesamtes für Verbraucherschutz und Lebensmittelsicherheit, LAVES, registration no. 10/0137).

#### 2.1. Hydrogel reservoir

Using a syringe, the NCO-sP(EO-stat-PO) solution was filled into the hollow silicone reservoirs (4.32 µL total volume). One end of the reservoir was equipped with a silicone septum which could be perforated with a tuberculin syringe with a needle diameter of 0.5 mm for sterile injection of DEX solution. To allow for a less traumatic implantation, each reservoir tip was smoothed down but remained unsealed in order to release DEX from the hydrogel. After being filled the reservoir was sterilized using ethylene oxide. DEX priming was performed during surgery immediately before reservoir insertion into the inner ear.

DEX sodium phosphate (Spectrum chemical MFG. Corp., Gardena, California, USA) was dissolved in PBS (PBS tablets, Invitrogen Corporation, Paisley, Scotland, UK). The DEX concentration in PBS was 50 µg/ µL for both HPLC and *in vivo* studies which necessitated a loading of 216 µg DEX per reservoir.

#### 2.2. Acoustically evoked auditory brainstem response measurement

Before surgery (day 0) and subsequently on day 3, 7, 14, 21 and 28, animals were anesthetized (diazepam 2.5 mg/kg p.o., atropine sulfate 0.05 mg/kg s.c., ketamine 30 mg/kg i.m., xylazine 7.5 mg/kg i.m.) in order to perform frequency-specific acoustically evoked auditory brainstem response measurements (AABR). All animals were treated with subcutaneous (s.c.) injections of carprofen (0.5 mg/kg), and atropine (0.05 mg/kg) for analgesia and reduction of secretion respectively.

Four needle electrodes were inserted s.c.: a positive pole at the vertex, a ground electrode in the neck, and negative poles postauricularily on the mastoids. The guinea pigs were placed in a soundproof box positioned on a heating pad with a temperature of 37°C. Speakers were connected to the acoustic meatus by calibrated pipette tips.

For frequency-specific acoustic stimulation and measurement, hardware and software from Tucker-Davis Technologies (Alachua, FL, USA) were used. Stimuli were 10 ms pure tones with a cosine-squared rise-fall time of 1 ms (24-bit Sigma-Delta D/A conversion at 200 kHz sampling rate). Six different frequencies (1, 4, 8, 16, 32, and 40 kHz) generated in 10 dB steps from 20 to 90 dB SPL (decibel sound pressure level) were presented using the TDT software (BioSigRP). The recorded neural signals were digitized at 24 kHz sampling rate (16-bit) and bandpass filtered between 0.3 to 3 kHz. The measurements of every frequency and sound intensity were recorded 270 times and averaged.

For evaluation purposes, data were exported into Microsoft Excel (Microsoft Corporation, Redmond, Washington, Seattle, USA) where the recordings were graphed and the triple standard deviation (SD) of background noise of each measurement, recorded in a phase of non-stimulation was calculated and plotted graphically. Hearing threshold was defined as the lowest stimulus level that generated a visually detected peak 3 exceeding three times SD. In the case where no hearing threshold could be detected at the systems limit of 90 dB SPL, the hearing threshold was set to 100 dB SPL. For every individual, frequency specific hearing thresholds of every measurement were compared to the related hearing threshold determined before surgery on day 0. The difference between the pre-surgical hearing threshold and those following was defined as hearing loss.

#### 2.3. Surgical procedures

Before surgery DEX solution and PBS were prepared freshly and filled into the reservoirs (for details see section 2.1.1).

Anaesthesia was performed similarly to that used for AABR but the dosage of ketamine and xylazine was higher (40 mg/kg i.m., respectively 10 mg/kg i.m.) and animals were supplied with enrofloxacine (10 mg/kg s.c.) for prevention of infection. Following initial AABR measurement on day 0 the postauricular area was bilaterally sheared placed under analgesia (1 ml prilocaine) and disinfected. Under sterile conditions a postauricular approach was performed to visualize the *Bulla tympanica*. The periosteum was abscised and the middle ear was opened with scalpel and forceps. Using an OP-MI microscope (Carl Zeiss AG, Oberkochen, Germany) the cochlea was identified and ventral to the round window a cochleostomy was drilled in the basal turn of the cochlea (drilling head 600 µm diameter). The reservoir was inserted 3 mm deep into the perilympathic space of the *Scala tympani* and left in situ. In an additional set of animals, reservoirs were inserted and withdrawn immediately to serve as a control trauma group. In all other groups the implant was fixed and the cochleostomy and fenestration of the middle ear were sealed with carboxylate cement (Durelon Carboxylate Cement, 3 M ESPE AG, 82229 Seefeld, Germany). Thereafter, the protruding part of the reservoir was rolled-up and secured s.c. and the wound was sutured in two layers.

Animals were treated bilaterally but some ears had to be excluded from the study due to e.g. reservoir displacement after tissue harvesting. To summarize the treatments, 11 animals (14 ears used) were treated with DEX administered by the hydrogel reservoir, 10 animals (12 ears used) received PBS released from the hydrogel reservoir and 7 animals (12 ears used) served as trauma group.

#### 2.4. Exploitation of specimens

On day 28 guinea pigs were anesthetised (ketamine 40 mg/kg i.m., xylazine 10 mg/kg i.m., atropine 0.05 mg/kg s.c.), the final AABR measurement was performed after which the sternal area of the animal was placed under analgesia with 4 ml prilocaine (2%). The chest was opened and the animal was perfused intracardially with 200 ml PBS and then fixed with 200 ml of modified Wittmaack fixing solution. The inner ears were extracted while the end section of the reservoirs remained inside the *Scala tympani*.

The *fenestra ovalis* and the apex were pierced with a lancet and the specimens were fixed in advanced Wittmaack fixing solution for up to 24 hours, rinsed for 10 hours with a 4% solution of lithium sulphate (Merck KGaA, Darmstadt, Germany) and dehydrated (2 hours per concentration) in ascending ethanol concentrations (50% v/v, 70% v/v 90% v/v and 100%) or optionally overnight in 70% v/v ethanol. Afterwards they were dried at room temperature and embedded in 5 parts epoxy resin and 2 parts hardening agent (SpeciFix-40 Kit, Struers GmbH, Ballerup, Denmark). Resin was adjusted by the addition of titanium oxide for whitening and reduction of transparency. Specimens and resin were filled into self-made silicone moulds with an inner diameter of 3 cm and a height of 4 cm (silicone source: SORTA Clear 40, Kaupo, Spaichingen, Germany) and placed into vacuum until all spaces in the specimens were filled with epoxy resin. The vaccum was slowly released and the samples were left to harden overnight at room temperature.

#### 2.5. Grinding, Histology

All specimens were grinded with a grinding machine (PowerPro 4000, Bühler, Lake Bluff, Illinois, USA) and abrasive paper. After reaching the cochlea with coarse sand paper (grain size 800) the process was then continued with fine sandpaper (grain size 1200). For every layer of the cochlea, 20 µm were abraded and the section was stained for two minutes with each eosine and toluidine (Merck KGaA, Darmstadt, Germany). The freshly stained surface of the specimen was photographically documented at 30-, 150- and 200-times magnification using a Keyence system (VHX-600 DSO, Osaka, Japan).

For assessment of tissue response inside the *Scala tympani*, Keyence software was used to measure the area occupied with connective tissue in the first and the last mid-modiolar plane section as well as that arithmetically lying in between those two sections. The 7 cross sections of the *Scala tympani* per section were titled as lower basal turn, upper basal turn, first middle turn, second middle turn, third middle turn, forth middle turn and apical turn [18]. Connective tissue span was set into a ratio to the area of the *Scala tympani* which were measured by tracking the inner outline of the Scala tympani using the Keyence software. Additionally, for every single layer and every part of the *Scala tympani* (lower basal to apical turn) a subjective evaluation using a ranking system was performed in order to monitor the distribution of connective tissue in relation to the point of cochleostomy and the turns of the cochlea. The rationale for subjective ranking was: score 0: no connective tissue; score 1: less than one quarter of the scala tympani is filled with connective tissue; score 2: less than half but more than one quarter of the scala has to be filled with tissue and/or the whole surface of the implants crosscut has to be covered; and score 3: more than half of the scala tympani is filled with connective tissue; see Fig. S1)

In order to correlate results from fibrosis evaluation and AABR measurements, averages of the single values of every group were composed and compared to the averaged results of hearing loss on day 28.

#### 2.6. Statistical Analysis

Statistical analysis was performed using GraphPad Prism 5 (GraphPad Software Inc., La Jolla, California, USA). Since animals were treated bilaterally, the correlation of left and right ears of same individuals were excluded, before using One-way ANOVA in combination with the Tukey post-test to compare mean hearing loss and measurement or ranking of tissue response within the turns for each group or for grouped comparison of tissue formation in the turns. Unpaired students t-test was used to calculate differences between results of mean tissue growth ranking and measurement between groups. Correlation assessment between tissue growth determined by ranking and hearing threshold shift was performed using a nonparametric correlation test (Spearman). Significance was defined as p-values with * = p<0.05, ** = p<0.01, *** = p<0.001.

## Results

### 3.1. *In vitro* results - Drug release studies from DEX loaded free form hydrogels

As reported previously [19], the free-form hydrogel reached the EWC state from dry state within 360 minutes and cryo-SEM pictures shows the macro-porous morphology of the hydrogel in the EWC state. (Fig. S2 and S2C). However, the pores are not inter-connected and the molecular mesh size of the hydrogel network remains decisive for diffusion of drug molecules through the hydrogel. The drug release from free standing 3D hydrogels is fully diffusion controlled and almost quantitative within 24 hours (Fig. S3).

### 3.2. *In vitro* results - Drug release studies from hydrogels in silicone tubes

**Figure 1:**
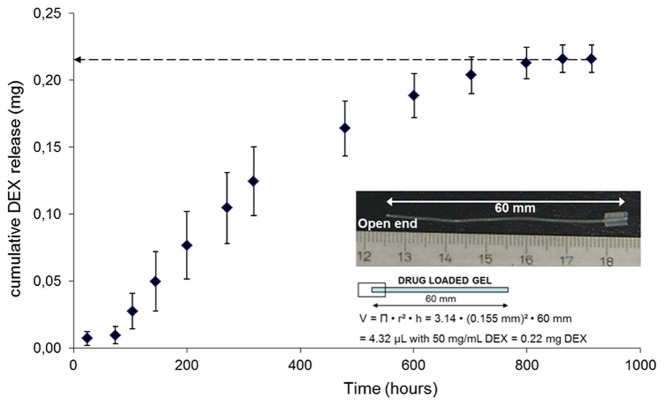
Image of a hydrogel reservoir with one end closed by a silicone septum for fluid injections (right end), while the other end is open in order to release the drug into the area to be addressed (left end) and calculation of the amount of drug inside the hydrogel filled tube. Drug release from this arrangement is shown in the diagram. The data points result from nine experiments from three different NCO-sP(EO-*stat*-PO) batches, each of them used for the preparation of three release setups.

In this configuration, the release of drug may logically occur only through the open end of the silicone tube. The walls of the silicone tube will restrict the hydrogel to swell to its full equilibrium state, hence at every point of time the hydrogel will exert a swelling force against the inner wall of the silicone tube by which the hydrogel adjusts tightly to the inside wall of the silicone tube. The exact dimension of the silicone tube reservoir with the amount of drug molecule inside the hydrogel and the cumulative release profile is shown in Fig. 1. Reproducibility of the hydrogel packing inside the silicone tube reservoir and the kinetics of the drug release were performed with three batches of separately prepared DEX loaded hydrogels with each of the batches inserted in three different tubes. Although the release of the drug happens by diffusion process, the geometrical constraint result in a time of about 900 hours or 38 days until DEX is quantitatively released. Interestingly, there was only negligible release in the first 50 hours. This may be explained by the initial diffusion of water inside the gel such that maximal water content in the tube is reached allowing the diffusion controlled release to start.

### 3.3. *In vitro* results - Drug release studies from hydrogel as diffusion gate for drug reservoir

**Figure 2:**
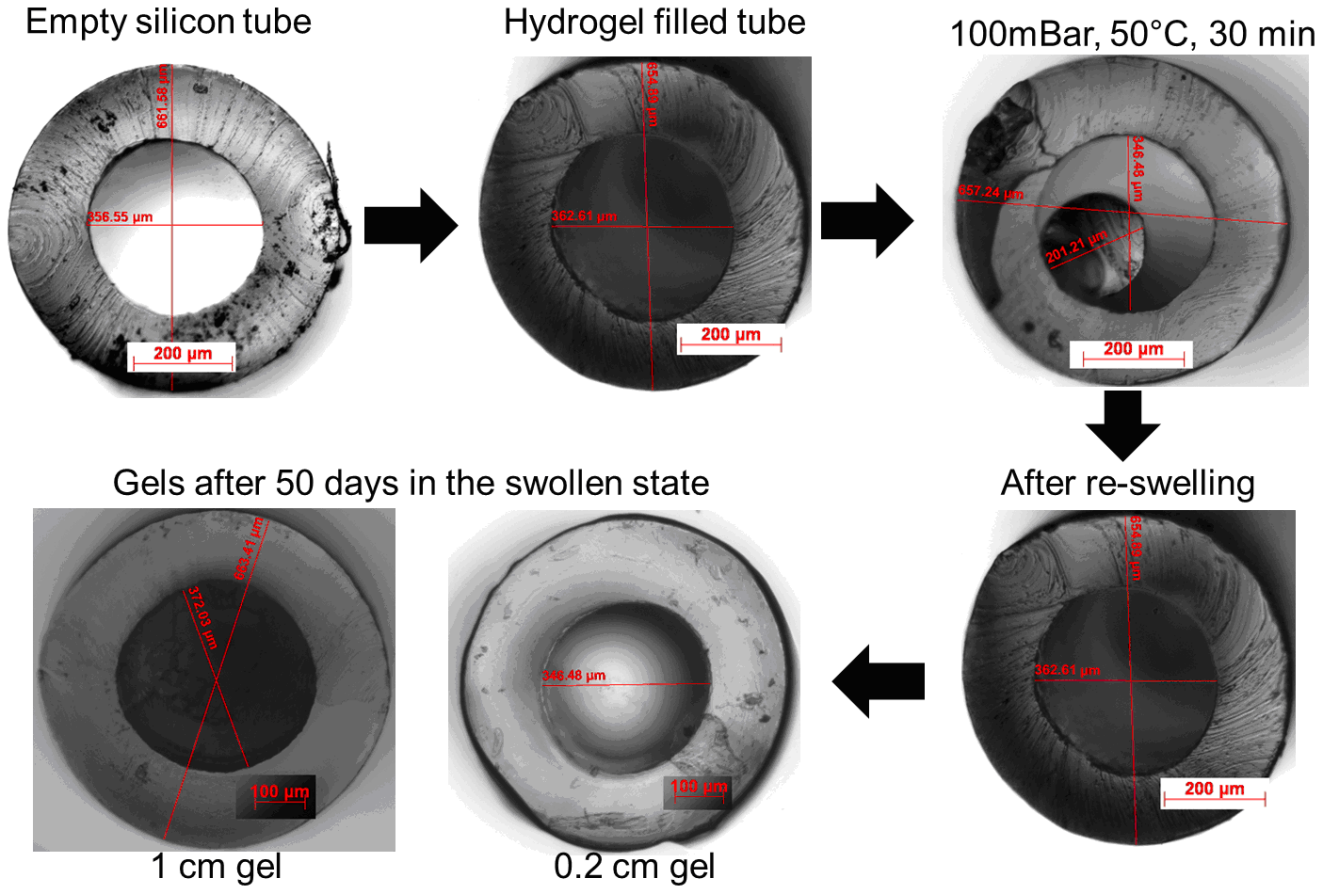
Cross-section images of silicone tubes before and after filling between 2 and 10 mm with with 20 mm with with 20% (w/v) NCO-sP(EO-stat-PO) gels (top left and middle), after drying of the gel (top right), re-swelling (bottom right) and after maintaining the gels in a swollen condition for 50 days (bottom left).

In this configuration, the open end of the silicone tube is filled hydrogel for a certain length, so as to create a diffusion gate that could possibly separate the inner ear from an externally accessible drug reservoir. We also evaluated the possibility to dry and re-swell the set-up and whether this would lead to a tight fit of the re-swollen gel inside the tubes. Fig. 2 presents a series of images showing cross-sections of silicone tubes before and after filling with gel. In order to evaluate whether the gels remain in the tubes after drying and re-swelling, the gels in the tubes were warmed to 50°C under reduced pressure (100 mbar) for 30 minutes which resulted in a strong shrinkage of the hydrogel. However, the gels remained at the place in the tubes where they had been placed and after re-swelling the gels tightly closed the inner lumen of the tubes without any visible gaps, this being independent of the hydrogel gate length.

We also checked whether longer incubation times affected the hydrogels within the tubes with regard to signs of degradation or morphological changes. Our results show that at least for 50 days, no changes of the hydrogel within the tubes could be observed.

We then prepared the aforementioned hydrogel gate arrangement in triplets and loaded the free space in the tubes with DEX solutions (100 mg/mL) to study the drug release kinetics. During this procedure (see Fig. S4 for details) it was necessary to create a pinhole at one place in the side wall of the silicone tube. In order to assess whether this pinhole did not result in uncontrolled release during the studies, we checked its tightness by using a dye solution (Fig. S5). This control experiment showed that the pinhole closes tightly after removal of the needle and no uncontrolled release occurs. Fig. S6 shows images of three samples with hydrogel gates of 10, 5 and 2 mm length as well as the calculation of drug loading and the drug release profiles. Again an initial lag-time was observed, however with about 100 hours being twice as long. This results from the need for DEX to diffuse through the hydrogel gates before release can occur. Quantitative release of DEX was reached in all three gate-length cases after approximately 1200 hours ( = 50 days).

Since DEX release started earlier in the completely gel filled tubes and the diffusion time of DEX out of those fully filled tubes remained for 38 days, which covers the critical time period in cochlear implant patients, these results encouraged us to examine whether this system would demonstrate beneficial effects *in vivo*.

### 3.4. *In vivo* results - AABR measurements

**Figure 3:**
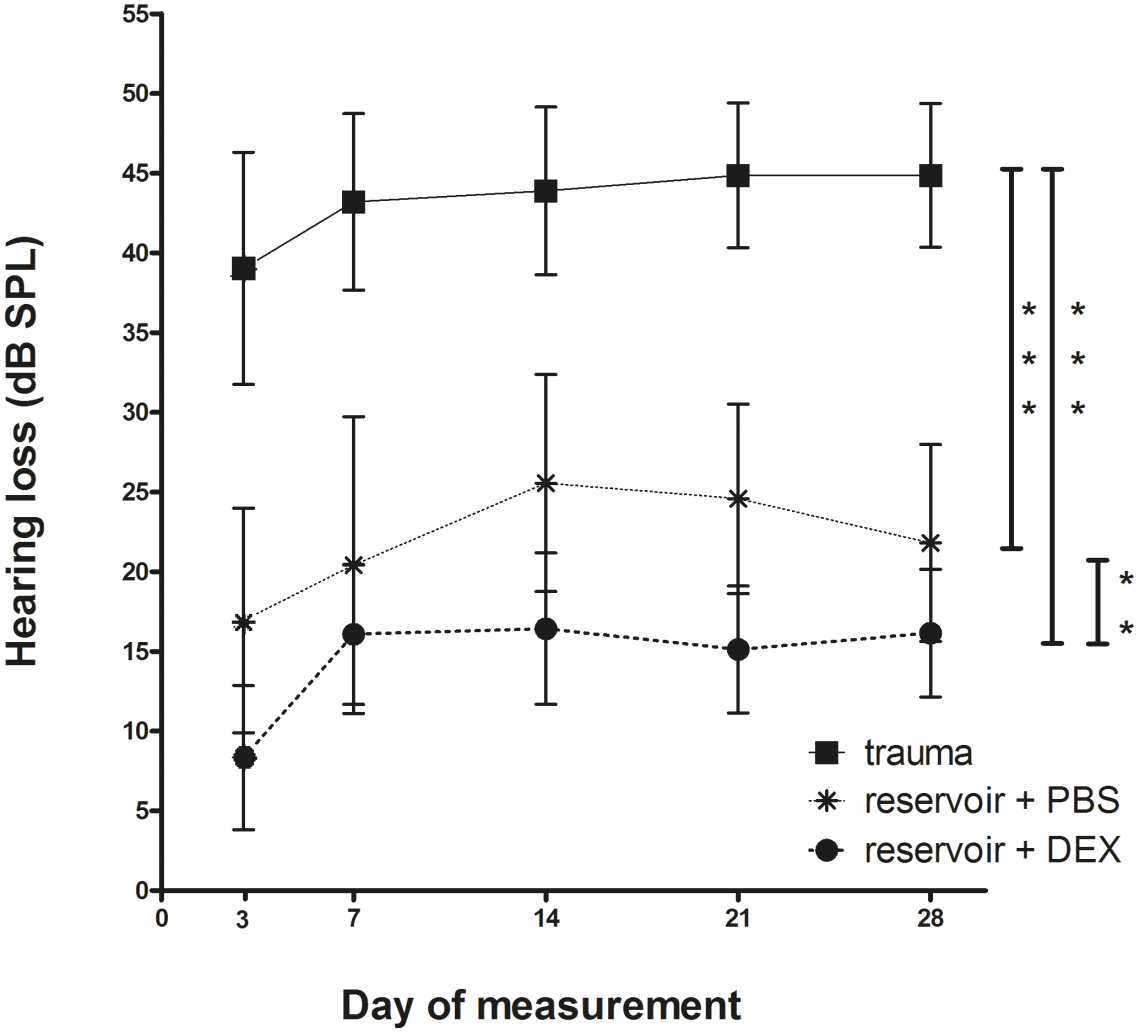
Mean and SEM of hearing loss (difference between the experimental days hearing threshold and hearing threshold before implantation) development from day 3 to day 28 for all experimental groups. Cochleae of the trauma group (n = 12) suffered from highest hearing loss compared to all other experimental groups. Additionally, individuals implanted with a PBS releasing reservoir (n = 12) lost hearing more significantly compared to the group having been implanted with the DEX filled reservoir (n = 14). The statistical differences using ANOVA test examined on experimental day 28 are plotted at the right side of the graph, demonstrating, that the reservoir + DEX treatment resulted in the significantly best hearing thresholds. (** = p<0.01; *** = p<0.001).

On experimental day 0, prior to surgery, all animals were possessing normal hearing. No significant differences in mean AABR thresholds, given as lowest sound intensity where the brainstem response exceeded the triple standard deviation of the background noise, were observed between experimental groups across all frequencies (data not shown). Postoperatively, all groups suffered from an initial loss of hearing detected in the average of all frequencies measured three days after surgery: the mean ± SEM difference compared to the day 0 threshold was: 8.33±4.52 dB SPL in the reservoir + DEX group, 16.94±7.04 dB SPL in the reservoir + PBS group and 39.03±7.28 dB SPL in the trauma group (Fig. 3).

This hearing loss stayed statistically stable within every group over the whole experimental time but it significantly differed among the four groups over this time period. Significantly better results in comparison to all other groups from day 3 until day 28 were detected in animals implanted with the reservoir applying DEX (hearing loss on experimental day 28: 14.42±1.54 (mean ± SEM) dB SPL; p_reservoir + DEX vs. trauma_<0.001, p_reservoir + DEX vs. reservoir + PBS_<0.01), whereas the highest hearing loss (43.17±1.08 (mean ± SEM) dB SPL) was measured in the trauma group (p_trauma vs. reservoir + DEX_<0.001, p_trauma vs. reservoir + PBS_<0.001) (Fig. 3)

Concerning the frequency specific hearing loss within each experimental group, differences are only seen between the 1 kHz and 8 kHz spectrum of the trauma group (hearing loss at 1 kHz: 28±4.55; at 8 kHz: 56.5±6.36 dB SPL (mean ± SD); p<0.05; Figure S7), or in the averaged results of all animals of all groups, where the mean hearing loss at 1 kHz was 15.94±3.35 SD dB SPL and thus statistically lower than hearing loss at 8 kHz (36.95±4.02 SD dB SPL, p<0.01), 16 kHz (31.47±4.22 SD, p<0.05) and 32 kHz (31.45±3.81 SD, p<0.05) (data not shown).

### 3.5. *In vivo* results - Tissue response

#### 3.5.1. Ranking of tissue growth – whole cochlea length

**Figure 4:**
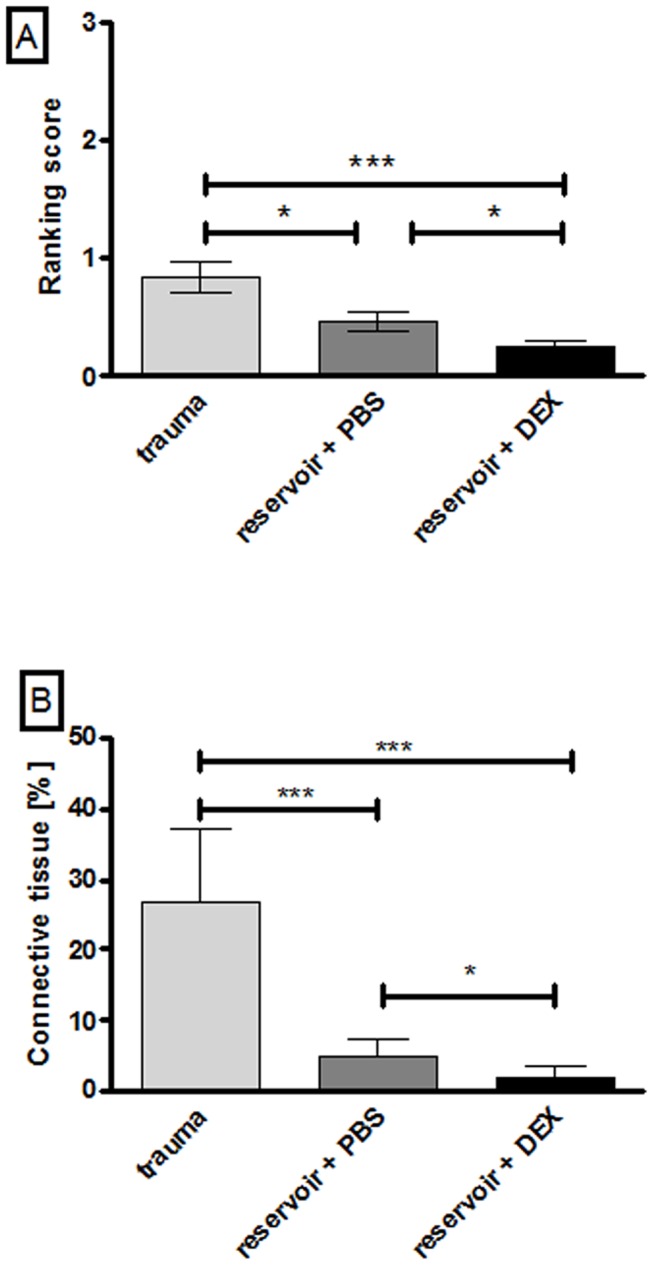
Graphed t-test results of tissue growth ranking (A) and measurement (B) for the whole cochlea length. Using both evaluation methods the tissue growth in the trauma group was significantly increased compared to the reservoir and DEX group with a p value <0.001. When applying the ranking score the difference between reservoir + PBS and trauma or reservoir + DEX was significant with p<0.05 (A). Comparing the tissue growth of the reservoir + PBS group with those of the trauma group or the DEX group using the measuring method, the p value is <0.001 (PBS vs. trauma) and <0.05 (PBS vs. DEX). Error bars: SEM. * = p<0.05, *** = p<0.001.

Using student's t-test the evaluation of fibrotic tissue response in the whole cochlea by use of a subjective scoring system revealed significant differences between the three experimental groups (Fig. 4A). The trauma group suffered from a more intense tissue response (score: 0.83±0.12 SEM) which can be statistically underscored with a p-value of 0.0133 when compared to the reservoir + PBS group, and p<0.001, when matched with reservoir + DEX. The group treated with the reservoir and PBS (0.47±0.08) also achieved a higher ranking score than the reservoir + DEX treated ones (0.25±0.05 SEM; p<0.05).

#### 3.5.2. Ranking of tissue growth - Distribution across scalae

**Figure 5:**
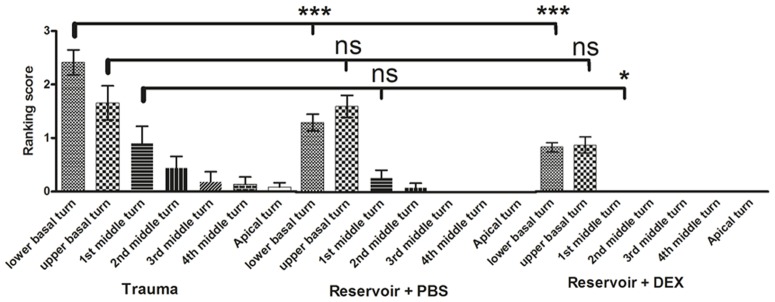
Distribution of fibrosis regarding subjective ranking for all inner ear turns and all experimental groups. In all groups the highest extend of connective tissue growth was detected in the basal turns. Fibroblast growth in more apical turns was only detected in the trauma group and did not take place in reservoir groups at all. Significant differences between the groups were detected (plotted above the SEM bars: * = p<0.05, *** = p<0.001; reference of the significance is marked by the thick bar) and were most prominent in the lower basal turns (p<0.001). Even though there seems to be a tendency of increased tissue formation in the PBS group compared to the DEX group there is no statistical relevance detectable using One-way ANOVA in combination with the Tukey post-test to compare the means between groups.

In all experimental groups tissue growth was significantly worse in the basal half turns of the cochleae (Fig. 5 and S8) compared to the apical region or the middle turns (p<0.001) (Fig. S8). Concerning this characteristic, all groups achieved similar tendencies. In the trauma group basal turns (lower and upper basal turn) achieved a ranking score of 2.04±0.25. Middle turns a score of 0.42±0.19 and apical turns a value of 0.08±0.08 (p<0.001; Fig S8A). Basal turns of the reservoir + PBS group showed tissue growth with a ranking score of 1.45±0.16 (Fig. S8B). Middle turns of 0.09±0.05 and basal turns of the reservoir + DEX group had a score of 0.88±0.11, while middle turns received a score of 0.0004±0.0004 (Fig. S8C). Both of the reservoir groups exhibited no fibrotic tissue response in the apical turns Comparison of fibrotic tissue reaction in middle and apical turns did not show any significant differences (Fig. S8A–C).

Differences between the experimental groups were also demonstrated most clearly in their disparity of tissue growth in the basal regions and the 1^st^ middle turn. Fig. 5, illustrating tissue growth ranking in all turns of all experimental groups, reveals the trauma group to undergo significantly more tissue growth in the basal turn (2.41±0.23 SEM) than both reservoir groups (p_trauma vs. reservoir+PBS_<0.001, p_trauma vs. reservoir+DEX_<0.001). The amount of newly formed tissue in the upper basal turns of all groups did not differ but the tissue in the first middle turn of the trauma group was significantly increased compared to the reservoir + DEX treated ones (p<0.05). In all scales more apical to this, no statistical relevant difference could be demonstrated among the groups and no significant differences were observed between PBS and DEX supported cochleae at all.

#### 3.5.3. Quantitative measurement of tissue growth-whole cochlea length

When measuring the area of fibrotic tissue growth and relating it to the matching area of the *Scala tympani* we detected the highest tissue formation in the trauma group where 26.78±4.17% (mean ± SEM) of the *Scala tympani* area was covered with fibrotic tissue or bony structures (Fig. 4B). In comparison to cochleae provided with the reservoir + PBS (4.74±1.11%) and reservoir + DEX (2.14±0.55%) the trauma control group suffered from significantly higher tissue proliferation (p<0.001). A relevant difference in tissue formation was detected in reservoir + PBS treated animals compared to DEX treated ones as well (p<0.05).

#### 3.5.4. Quantitative measurement of tissue growth – Distribution across scalae

**Figure 6:**
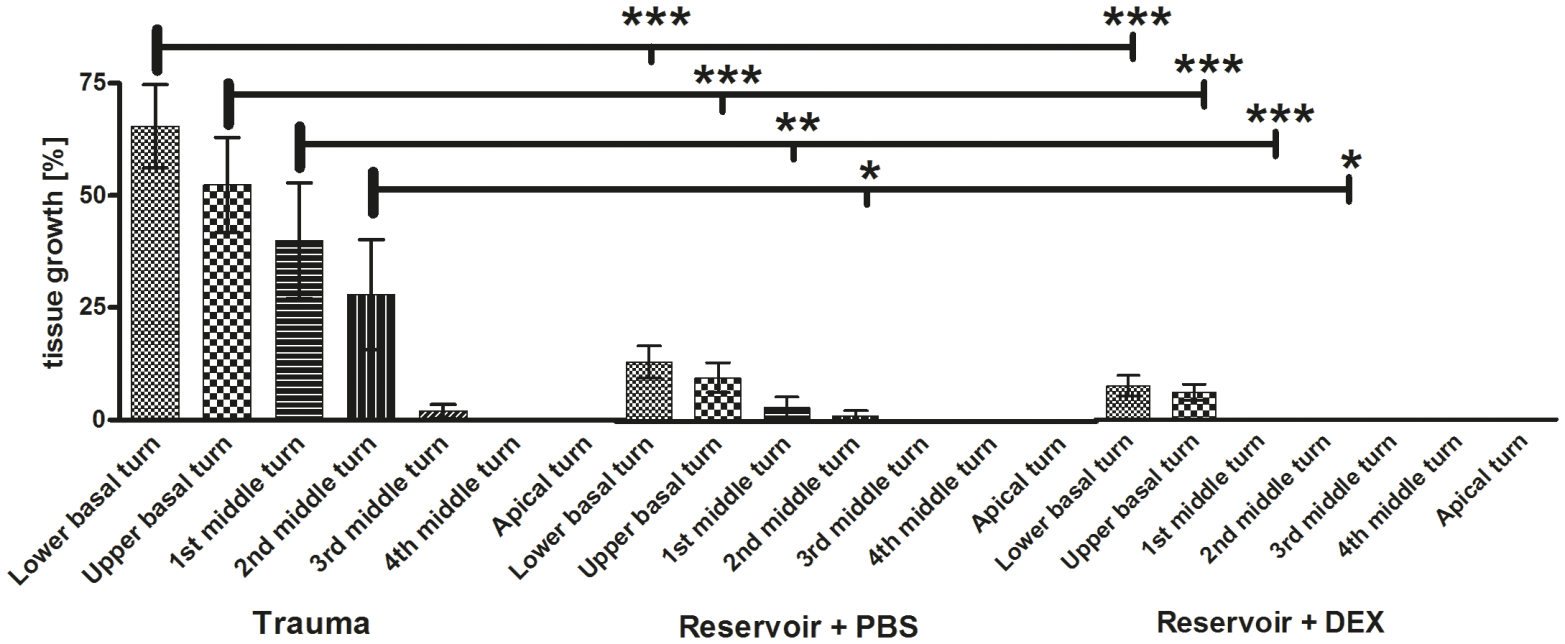
In this graph the mean and SEM percentage of measurement of connective tissue growth in the scala tympani of all experimental groups for each cochlear turn from basal to apical are plotted. The comparison of tissue growth between the groups is illustrated by horizontal lines. Reference of the significance is marked by the thick bar. Highly significant differences were observed between tissue growth in the trauma group and groups receiving PBS or DEX by reservoir. No differences were measured between PBS or DEX treated groups even though a slightly increased connective tissue growth seems to be apparent in the PBS group compared to DEX treated animals. In cochleae implanted with a PBS releasing reservoir tissue growth could be measured from basal up to the 2^nd^ middle turn whereas in the reservoir and DEX treated cochleae connective tissue formation was only visible in the lower and upper basal region. One-way ANOVA in combination with the Tukey post-test was used to compare means between groups: * = p<0.05; ** = p<0.01; *** = p<0.001.

The significantly most affected location of the cochlea are the basal turns when compared to more apical regions (p between <0.05 and <0.001; Fig. S9A–C). Fig. 6 and Fig. S9 illustrate the decrease of tissue growth from the basal to the apical parts of the cochleae. Comparisons of tissue growth within the turns of each experimental group are plotted in Fig. S9 A–C.

Additionally, the groups showed different amounts of tissue in the individual parts of the cochlea. In the lower basal turn trauma group reached a tissue fraction of mean ± SEM of 65.41±9.32% with a p-value of <0.001 compared to the tissue response found in the lower basal turns of reservoir + PBS group (16.17±4.01%), and reservoir + DEX group (8.25±2.46%) (Fig. 6). Similar findings are seen in the upper basal turn where the trauma group suffered from a tissue growth of 52.36±10.64%, and thus a statistically higher tissue reaction than the reservoir + PBS group (11.86±3.88, p<0.001) and reservoir + DEX group (6.67±1.95%, p<0.001). In the 1^st^ middle turn the differences are found in p-values <0.01 between trauma group with a score of 39.95±12.92%, and reservoir + PBS group with a score of 3.7±2.91% and <0.001 for the reservoir + DEX group with 0.05±0.05%. Evaluation of the 2^nd^ middle turn reveals a p-value of <0.05 for the difference between the trauma group (27.87±12.27%) and reservoir + PBS group (1.43±1.43%) and the reservoir + DEX (0%). In the 3^rd^ middle turn of the trauma group 1.83±1.59% of the scala tympani area was filled with tissue, which was compared to the 3^rd^ middle turns of the PBS or DEX treated groups, where no tissue growth was observed at all, although this tissue reaction was not statistically relevant. In all turns more apical to this point, no statistical distinction was found since no tissue growth was detected apical to the 3^rd^ turn in any of the experimental groups (Fig. 6). Comparing the tissue growth measured in PBS treated animals to those in DEX treated ones no statistically relevant difference was observed even though the tendency of higher fibrosis rate in PBS treated animals (lower basal: 16.17±4.01%; upper basal 6.67±1.95%; 1^st^ middle 3.70±2.91%; 2^nd^ middle: 1.43±1.43%) compared to DEX treated guinea pigs (lower basal: 8.25±2.46%; upper basal 11.86±3.88%; 1^st^ middle 0.05±0.05%; 2^nd^ middle: 0%) (Fig. 6) was clearly seen.

#### 3.5.5. Differences in ranking and quantitative measurement

Results from subjective ranking of tissue reaction in all images taken from the scala tympani of all experimental animals and the findings from the tissue growth measurement performed in three pictures of each cochlea are very similar but significances differed between both methods when comparing the mean values of the experimental groups. More precisely, ranking showed a p-value of <0.05 for the difference between the trauma and the reservoir + PBS group (Fig. 4A), which is p<0.001 according to the measurement technique (Fig. 4B). Next to this, the statistical results were equal.

#### 3.5.6. Correlation of tissue growth and hearing impairment

**Figure 7:**
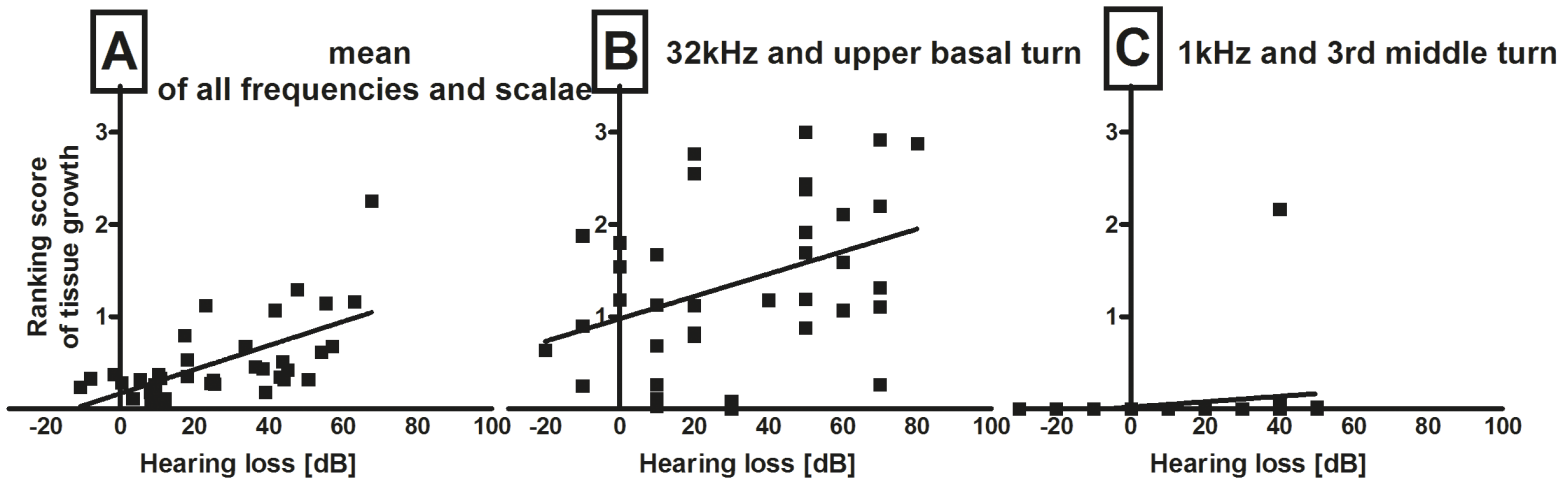
Correlation of hearing loss measured on day 28 and tissue reaction determined by ranking. Graph A illustrates that with the amount of tissue reaction evaluated over the whole cochlea length the loss of residual hearing in all frequencies increases (p<0.001; r = 0.6338). This effect is detectable in lower and higher frequency regions of the cochleae as well. In figure 7B the detected tissue reaction and hearing loss at the upper basal turn and at 32 kHz are plotted as an example for the correlation in higher frequency areas 7B the detected tissue reaction and hearing loss at the upper basal turn and at 32 kHz are plotted as an example for the correlation in higher frequency areas kHz are plotted as an example for the correlation in higher frequency areas (p<0.05; r = 0.3742). Fig. C is an example for correlation of tissue reaction and hearing loss in lower frequency areas of the cochlea. Here we correlated hearing loss at 1 kHz and tissue growth in the 3 kHz and tissue growth in the 3^rd^ middle turn (p<0.05; r = 0.3727).

Tissue growth concurred with the results of AABR measurements. A profound correlation between mean values of the amount of tissue, determined by subjective ranking, and the averaged hearing loss of all frequencies of the individual animal was ascertained. It was demonstrated that with increasing tissue growth hearing loss increased (Fig. 7A). Using the Spearman-Rho-tests a correlation with r = 0.6338 (p<0.001) was detected.

Correlation between the location of new tissue formation in the scala tympani and hearing loss at specific frequencies was detected as well, that is to say, accumulation of tissue growth in basal parts of the cochlea were associated with loss of hearing in high frequencies, equally, tissue formation in the more apical cochlear turns did accompany loss of hearing in. lower frequencies. For example, the fibrous tissue expanse in the upper basal turn of all evaluated cochleae corresponded significantly (p = 0.0225) to the hearing loss detected at 32 kHz (r = 0.3742). And the other way around, the amount of tissue detected in the 3^rd^ middle turn correlated significantly to the hearing loss at 1 kHz (p = 0.00231; r = 0.3727 (Fig. 7B and C).

The trauma group, which showed almost no residual hearing on day 28 (hearing threshold: 93.75±2.24 dB SPL) and thus the most severe hearing loss (44.86±4.52 dB), was marked by the most intense new tissue formation especially in the lower basal turn of the cochlea (ranking score for whole cochlea: 0.83±0.12; measurement: 26.78±10.21%). Animals provided with the reservoir plus PBS showed less tissue growth (ranking: 0.47±0.08; measurement: 4.74±2.49%) and reduced hearing loss (21.81±6.18 dB) and cochleae that were treated with the DEX reservoir developed minimal fibrotic outgrowth (ranking score: 0.25±0.05; measurement: 2.14±1.38%) associated with a minimal loss of hearing ability 28 days after implantation (16.15±4 dB).

#### 3.5.7. Angiogenesis and osteogenesis

Additionally to fibrotic tissue growth, an organisation of the tissue in terms of angiogenesis was detected in 5 cochleae of the trauma group and one ear of the reservoir + PBS group. Furthermore, in two cochleae of the trauma group initial signs of osteogenesis were visible (see Fig. S1D).

## Discussion

The aim of this study was the evaluation of a novel hydrogel reservoir for inner ear drug delivery *in vitro* and *in vivo*. The hydrogel precursor NCO-sP(EO-*stat*-PO) possesses highly reactive isocyanate (NCO) groups which can potentially react with the alcohol groups of DEX. However, the presence of the phosphate groups and the two sodium counter ions render the molecule extremely hydrophilic while the isocyante groups are placed at relatively hydrophobic iosphorne-rings. Moreover, the hydroxyl groups of the drug molecule are directly attached to the aromatic ring. These various properties prevent permanent covalent binding of DEX to the hydrogel network to any detectable level [19].The free form hydrogels exhibited the fastest release kinetics within 24 hours as the release medium could have access to all the 3D surface of the hydrogels by which the diffusion of entrapped drug molecules in all the direction. The drug release was by swelling controlled diffusion process. The system where the hydrogel was completely filled inside the silicone tube, only the open end of the silicone tube have access to the release medium to the hydrogel. In this configuration, the wall of the silicone tube restricted the swelling kinetics of the hydrogel. In this system, the drug release was purely by diffusion controlled process which took almost 900 hours to release the loaded drug completely. In the other system where, the hydrogel without any drug in it was only acting as the diffusion gate, the drug from the reservoir had to diffuse through the hydrogel gate to reach the release medium; it took almost 1200 hours to release the drug completely. In the 1200 hours, the initial 100 hours was the lag time during which the drug had to diffuse through the gel gate. All these three configurations explain how the release of the drug can be controlled precisely by checking the access of the release medium to the hydrogels. Finally, examination of the hydrogel as a drug delivery matrix in a medical device used for inner ear treatment *in vivo* was carried out.

DEX was chosen for this drug delivery study as model drug because of its well-known anti-inflammatory and hair cell protective effects [6], [7], [20]–[22]. DEX suppresses apoptotic activities in response to TNF-α. Studies performed by Messmer and colleagues demonstrate that this effect can be achieved even up to 12 hours after TNF-α application. DEX retards the down-regulation of inhibitors of apoptosis proteins [23], blocks transcription of pro-inflammatory molecules and increases transcription of anti-inflammatory factors and reduces leukocyte migration through decreasing the adherence of these cells to the vascular endothelium [24]. Synthetic glucocorticoids are also able to decrease vascular permeability and vasodilatation, prominent features of the inflammatory reaction and necessary for the emigration of immune cells. Consequently, glucocorticoids reduce oedema and fibrin formation, inhibit capillary and fibroblast proliferation and enhance collagen breakdown [24].

These effects play an essential role in the neutralisation of foreign bodies or bacteria raising the consideration regarding the use of DEX in cases where infection may be likely or is actually present. Conversely, all of these properties are necessary in the suppression of inflammation concerning cochlear implantation. Hence, although a precautious use of DEX is essential, it is a noteworthy ally for offering protection of hearing ability following cochlear implantation [25]–[27].

The extent of tissue response was determined both by ranking and direct measurement. The results from both the methods were not equal but did tend to deliver the same information. While the measurement of 3 slices per scale per cochlea brought the quality of objective evaluation, the subjective ranking of tissue growth in every single slice covered a quantitative assessment. Since the measurement was performed on 1 mid-modiolar image and the one directly before and one directly after the modiolus, the measured area was identical in all cochleae. This leads to a high comparability between the cochleae but also implies that alteration could only be detected in this specific region. Although the ranking underlay a subjective assessment, it was exclusively performed by one person who obliterates variation due to diverging assessment criteria of various individuals. In addition, scoring between 0 and 3 only allowed for an unrefined classification although as a positive factor, it involves the whole cochlea. As such, it is considered that the subjective ranking of all slices leads to a more comprehensive and accurate estimate of the level of tissue reaction.

Probably all distinctions between ranking results and measurement results concerning p-values are based on the alteration of modiolus-near or modiolus-distant evaluation and the fact that three images per scale used for objective measurement may not give a synopsis on the status of the whole cochlea. Nevertheless, ranking and measurement methods led to similar results in most cases, indicating that measurement can be used to support the coarser ranking score with more detailed values.

The reservoirs remained inside the *Scala tympani* and interacted with the inner ear in terms of foreign bodies over a time period of four weeks. Nevertheless, AABR results and the distribution of tissue growth along the cochlea length have shown that producing a trauma as performed on animals in the trauma group, has a more devastating influence on the structures of the inner ear. An initial hearing loss after surgery was observed on day 3 which remained stable over time. We consider that this initial hearing loss is brought about by the implantation procedure as well as the physical presence of the implant itself in the *Scala tympani* which may modify the fluid and basilar membrane movement and induce inflammatory reactions. An additional reason may be due to mechanical damage of hair cells although this would occur only in the basal region of the cochlea where the implant is located and would consequently affect only the higher frequency regions. Due to the fact that no differences in hearing loss between lower and higher frequencies were observed, this seems unlikely.

In contrast to Braun and colleagues who reported DEX-dependent hearing preservation without tissue reduction [28], we observed a correlation of hearing ability and fibrosis which has recently been reported by O'Leary [29]. These parameters depend on the same factor, inflammation, which lends to the concept that the tissue growth modifies the influence of the travelling wave towards the basilar membrane which in turn reduces exhibition of hair cells. Reduction of the nutrition requirement of sensory cells in this area may also occur as a consequence. Additionally, tissue proliferation inside the *Scala tympani* may disturb the travelling wave on its way towards the apical turns and consequently prohibit the stimulation of more apical sensory cells. As a result of this, hearing of higher frequencies is reduced as well as the sensation of low frequencies albeit the inflammation bring localised mostly in the basal turns.

All animals of the trauma group suffered from significantly increased hearing loss and tissue reaction compared to the other experimental groups. We believe that the double movement of the reservoir inside the cochlea of trauma group specimens, insertion as the first manipulation and explantation as the second, is a valid reason for this finding. In contrast, cochleae of the other groups had to sustain only one traumatic manipulation.

These results indicate that common application aids that have to be removed after usage may lead to traumatic changes of the inner ear. Consequently we suggest that an application aid, at least if its properties are comparable to the hydrogel reservoirs tested in this study, should be bound to the CI and remain inside the cochlea. Further studies concerning this topic would be necessary to evaluate if the findings from this study are generally representative for removable drug delivery systems. In addition to the different mechanical stimuli, it is important to consider whether more immunologically active cells arrived from the blood into the *Scala tympani* after the necessary rupture of the *Stria vascularis* during cochleostomy in cochleae of the trauma group. Although the opening of the cochleostomy was filled with carboxylate cement, it was not blocked with a close-fitting implant like the silicone reservoir and as a result the blood vessels inside the *Stria* may not have been compressed. Additional irritation by the cement may be assumed with respect to reports concerning pulpa irritation in dental medicine [30], [31] and to a newly published study stating that dental cement, applied on guinea pig inner ears, leads to a strong new bone formation [32], even though this method is a widely used [33], [34] and other reports have also assessed carboxylate cement to be non-irritating [35], [36].

Luttikhuizen and colleagues stated in 2006 that inflammatory processes are related to components of the blood stream; not only immune cells like macrophages but, for example, proteins like fibrinogen, the complement system, antibodies and various inflammatory factors [37]. They are attracted by chemotactic factors that are secreted from injured tissue or by cells stressed by the presence of foreign bodies [4], [38]. Although materials of current implants are biologically inert, they still trigger inflammatory responses of the surrounding tissue. This is based upon endogenous proteins that attach to the surface of the implant and attract components of the nonspecific immune system. In the later stages of the inflammatory process, following earlier leucocytic invasion, fibroblasts migrate towards the implant and build a tissue coating around the foreign material in order to protect the healthy tissue. This step is clearly seen in many specimens with implanted reservoirs (see Section 3.2.2).

Angiogenesis, another cardinal sign of inflammation, is seen in cochleae of the trauma and reservoir + PBS group. In cochleae of the trauma group angiogenesis had already progressed into a stage of osteogenesis. Subsequent to fibrotic tissue growth, angiogenesis follows as a consequence of the coagulation cascade and hypoxia and is incited by locally released histamine and fibrin fragment E [37]. Since most of these cells and substances are bound to the blood stream, the correlation between the effluence of blood into the cochlea and higher levels of inflammation must be considered.

Furthermore, the diameter and related rigidity as well as the material composition of the inserted piece of the reservoir, played an accessory role for the severity of the inflammatory reaction. Previous reports by Jolly and colleagues [26] demonstrated that electrode trauma depends on the size and flexibility of the array and that reduction of trauma can be achieved by flexible implants that easily adapt to the angulations of the cochlear turns. This information can be transferred to the implantation of silicone reservoirs that appear to be flexible which according to Jolly [26] should provoke less irritation in the tissue of the cochlea. This depends strongly on the composition of the material. The process of implantation and fixation of the reservoirs explains similarities between the impact administrated by reservoirs with either PBS or DEX. Nevertheless, significant higher fibrosis in PBS treated ears discovered over the whole length of the cochleae and the distinct angiogenesis in these cochleae prove the influence of DEX applied via the reservoir.

## Conclusions

In this study, the technical feasibility of incorporating hydrogels prepared from NCO-sP(EO-*stat*-PO) pre-polymers within the silicone tube for sustained release of DEX for several weeks through diffusion process into the inner ear was demonstrated which encouraged the *in vivo* studies.

Animal experiments using normal hearing Dunkin Hartley guinea pigs followed by treatment and regular measurement of hearing loss showed significantly lower fibrosis in case of DEX loaded hydrogel reservoir as compared to a PBS releasing control-reservoir and pure trauma. Most importantly, the hearing loss was significantly lower in case of the DEX loaded hydrogel reservoirs as compared to the other groups. In contrast, considerable functional and morphological changes were detected in the trauma group. We hypothesize that the insertion and immediate explantation of a delivery device is even more destructive to the inner ear than the impact of a foreign body in terms of a reservoir.

Our results strongly suggest that the NCO-sP(EO-*stat*-PO) hydrogel reservoir is a promising drug delivery device to apply water-soluble drugs in therapeutically relevant doses into the inner ear for a sustained treatment period. We conclude that the hydrogel used in combination with a rechargeable design of reservoir is a promising alternative of drug delivery device. This method might be optimized by adapting the reservoir to the cochlear implant in order to combine its insertion with the inevitable implantation of the CI, and thus avoiding additional trauma. This would allow surgeons to decide on the execution of drug application during surgery.

## Supporting Information

Figure S1 **Representative images of tissue response scores A) representatively depicts the score 0 mainly detected in animals of the reservoir + DEX group.** Images B) and C) illustrate score 1 and 2 representative for the reservoir + PBS or trauma groups. Score 3 is shown in D) which is taken from an animal of the trauma group. In this figure the reorganization of fibrotic tissue response (black arrow) in terms of ossification (white arrow) is clearly seen. Asterixes: hydrogel; black arrow head: silicone reservoir, missing in image D), taken from the trauma group, where the tubing was implanted and subsequently explanted.
(TIF)

Figure S2 **Relevant chemical structure (A) Hydrogel precursor and the chemical reaction of isocyanates with H_2_O, (B) shows the swelling kinetics of the hydrogel from its dry state to its fully swollen state, (C) microporous structure of the swollen hydrogel.**
(TIF)

Figure S3 **Structure of dexamethasone 21-phosphate disodium salt (DEX) and its complete release profile from the free form hydrogel.**
(TIF)

Figure S4 **Experimental procedure for sample preparation of the release studies from the silicone tubes with hydrogel gates.**
(TIF)

Figure S5 **Control experiment using a dye-solution showing that the pin-hole created in the silicone tube during loading of the DEX solution does not result in uncontrolled release.**
(TIF)

Figure S6 **Picture of hydrogel-gates with three different lengths in silicone tubes (top left), calculation of the amount of DEX in each of the tubes (top right) and result of the release studies (bottom).**
(TIF)

Figure S7 **Frequency specific hearing loss.** The mean and SD of hearing loss of all experimental groups after 28 days of implantation is plotted for each frequency tested. In all groups the hearing loss seems to be less affected in the lower frequencies but statistical evaluation did not show any significant differences between the frequency specific hearing loss in any of the experimental groups.
(TIF)

Figure S8 **Tissue growth evaluated by ranking.** The mean ± SEM results of subjective ranking of tissue formation in the cochlea turns of each experimental group are plotted. In all groups the tissue reaction is significantly increased in the basal regions compared to the middle and apical regions. Fibrotic tissue response in more apical turns was only detected in the trauma group (A) and did not take place in reservoir groups treated with PBS (B) or DEX (C). One-way ANOVA in combination with the Tukey post-test was used to compare the tissue growth within the different cochlea turns of each experimental group: ** = p<0.01; *** = p<0.001. Reference of the significance is marked by the thick bar.
(TIF)

Figure S9 **Tissue growth evaluated by measurement.** The mean ± SEM percentage of scala tympani area of each cochlea turn covered with tissue is plotted for each experimental group. In all groups the tissue reaction is significantly increased in the basal regions compared to the middle and apical regions. Fibroblast growth in more apical turns was only detected in the trauma group (A) and did not take place in reservoir groups treated with PBS (B) or DEX (C). One-way ANOVA in combination with the Tukey post-test was used to compare the fibrous tissue growth within the different cochlea turns of each experimental group: ** = p<0.01; *** = p<0.001. Reference of the significance is marked by the thick bar.
(TIF)
