# Supplementary figures and images for: In Vitro and In Vivo Evaluation of a Hydrogel Reservoir as a Continuous Drug Delivery System for Inner Ear Treatment
Source: PLoS One. 2014 Aug 8;9(8):e104564. doi: 10.1371/journal.pone.0104564 (PMC4126769; doi:10.1371/journal.pone.0104564)

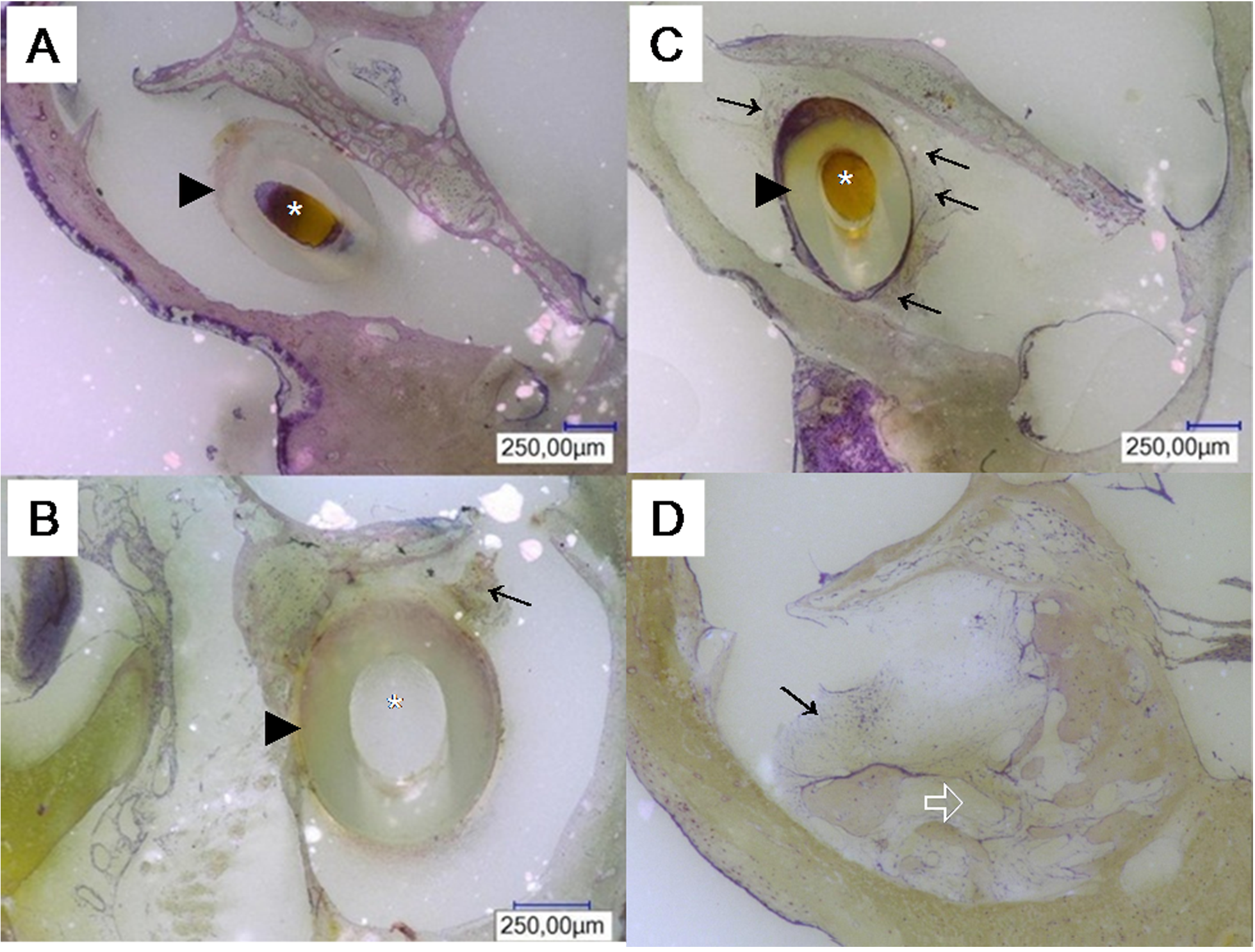

Supplement: Figure S1 — Representative images of tissue response scores A) representatively depicts the score 0 mainly detected in animals of the reservoir + DEX group. Images B) and C) illustrate score 1 and 2 representative for the reservoir + PBS or trauma groups. Score 3 is shown in D) which is taken from an animal of the trauma group. In this figure the reorganization of fibrotic tissue response (black arrow) in terms of ossification (white arrow) is clearly seen. Asterixes: hydrogel; black arrow head: silicone reservoir, missing in image D), taken from the trauma group, where the tubing was implanted and subsequently explanted. (TIF) [file pone.0104564.s001.tif]

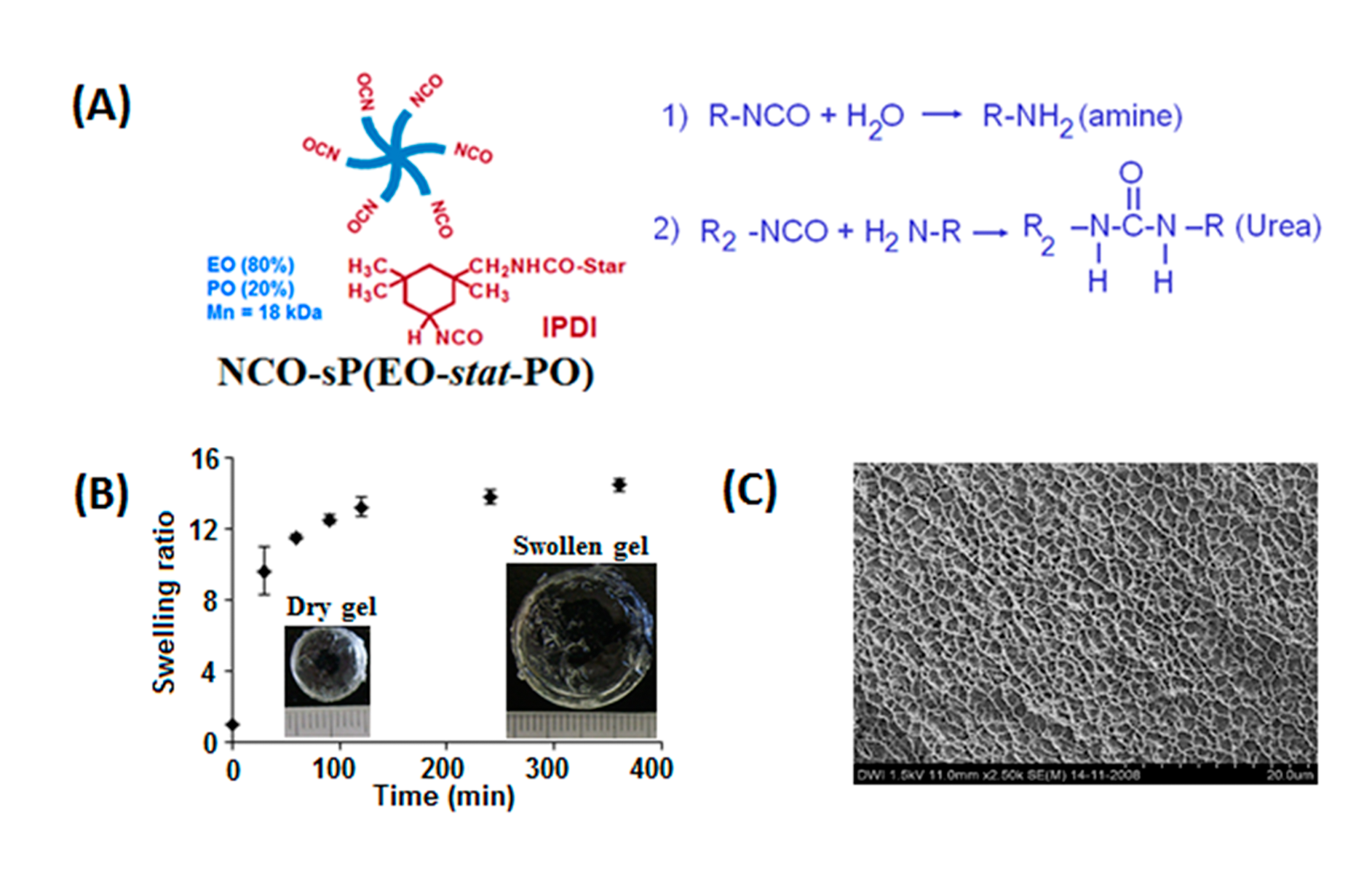

Supplement: Figure S2 — Relevant chemical structure (A) Hydrogel precursor and the chemical reaction of isocyanates with H2O, (B) shows the swelling kinetics of the hydrogel from its dry state to its fully swollen state, (C) microporous structure of the swollen hydrogel. (TIF) [file pone.0104564.s002.tif]

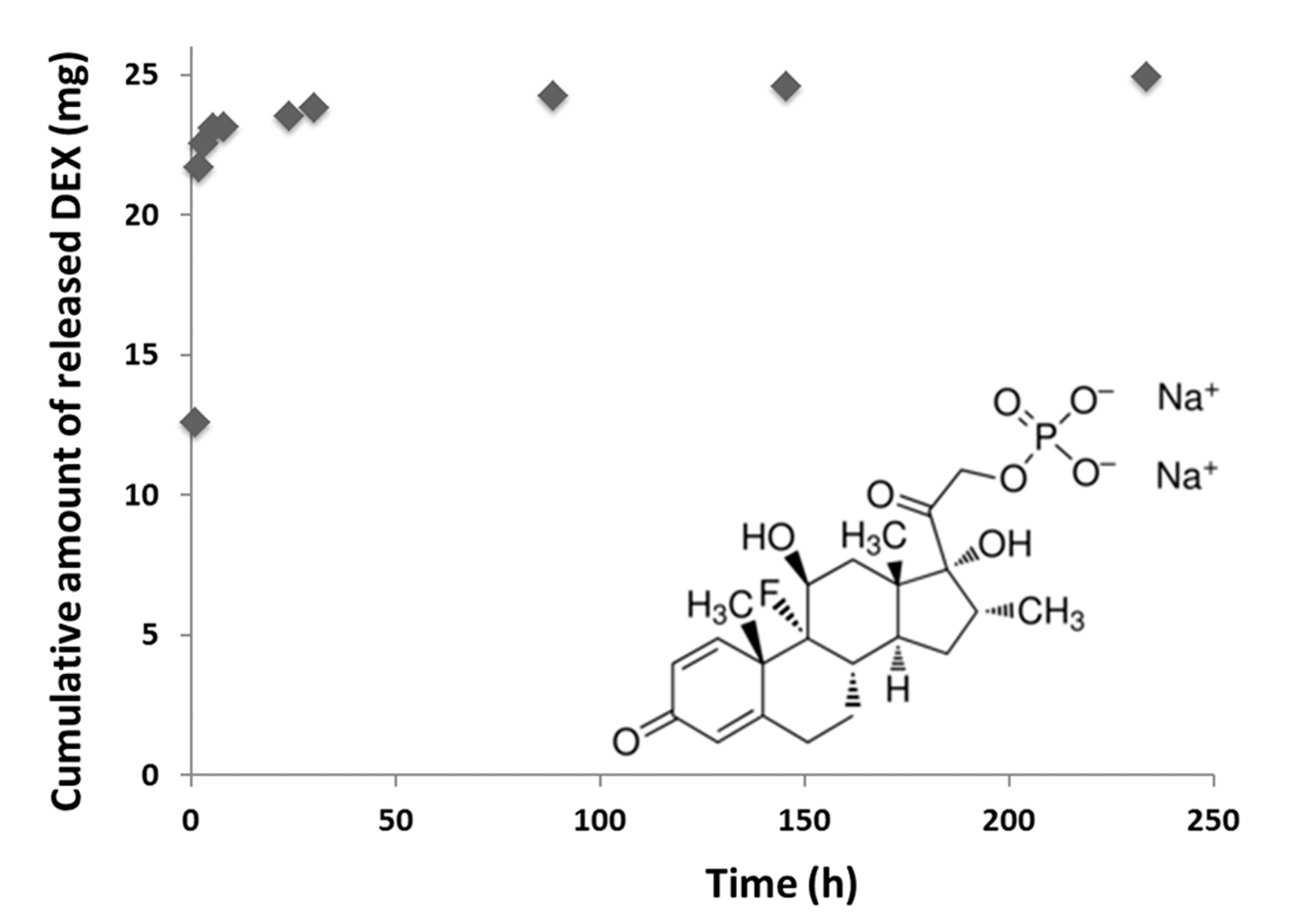

Supplement: Figure S3 — Structure of dexamethasone 21-phosphate disodium salt (DEX) and its complete release profile from the free form hydrogel. (TIF) [file pone.0104564.s003.tif]

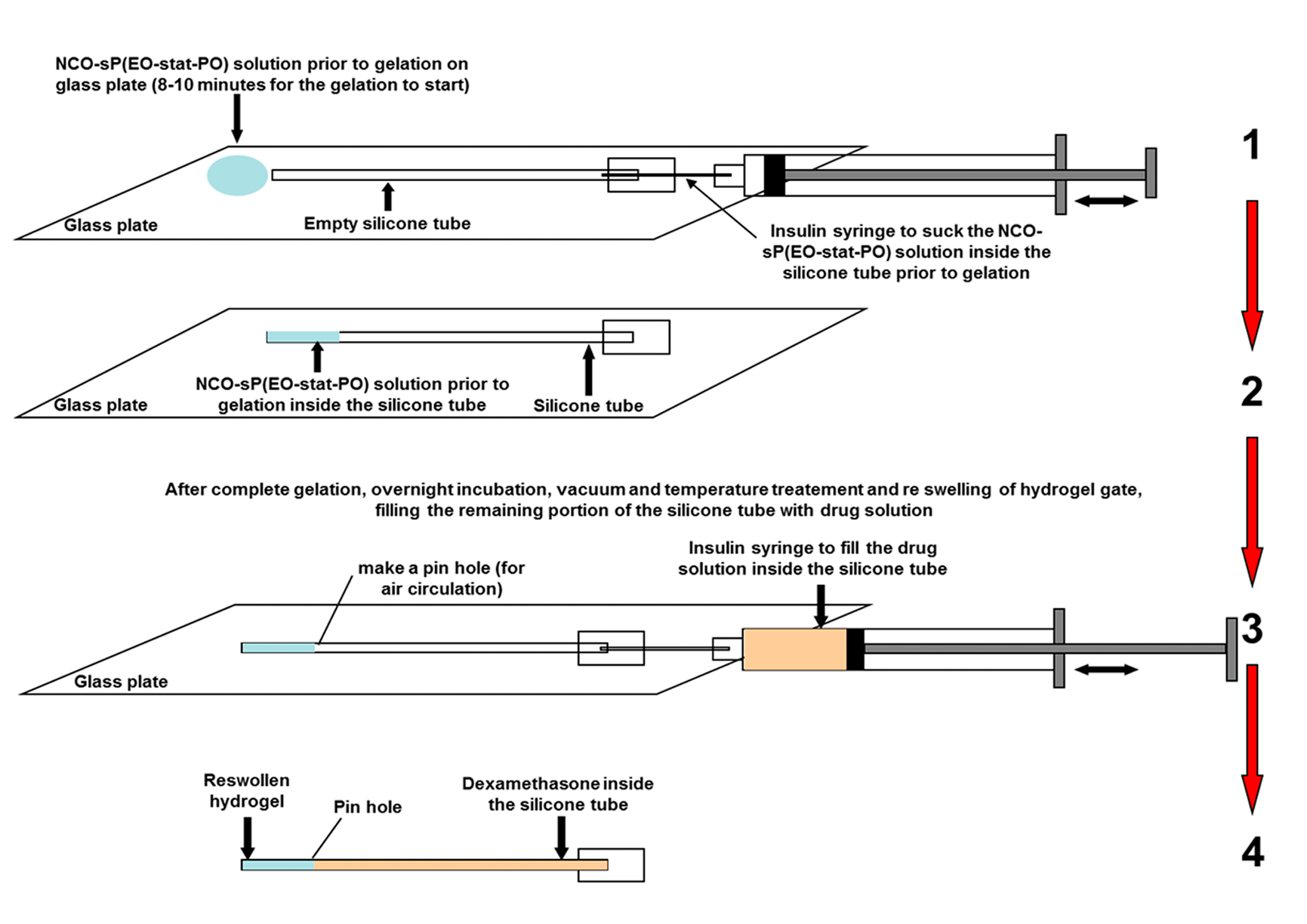

Supplement: Figure S4 — Experimental procedure for sample preparation of the release studies from the silicone tubes with hydrogel gates. (TIF) [file pone.0104564.s004.tif]

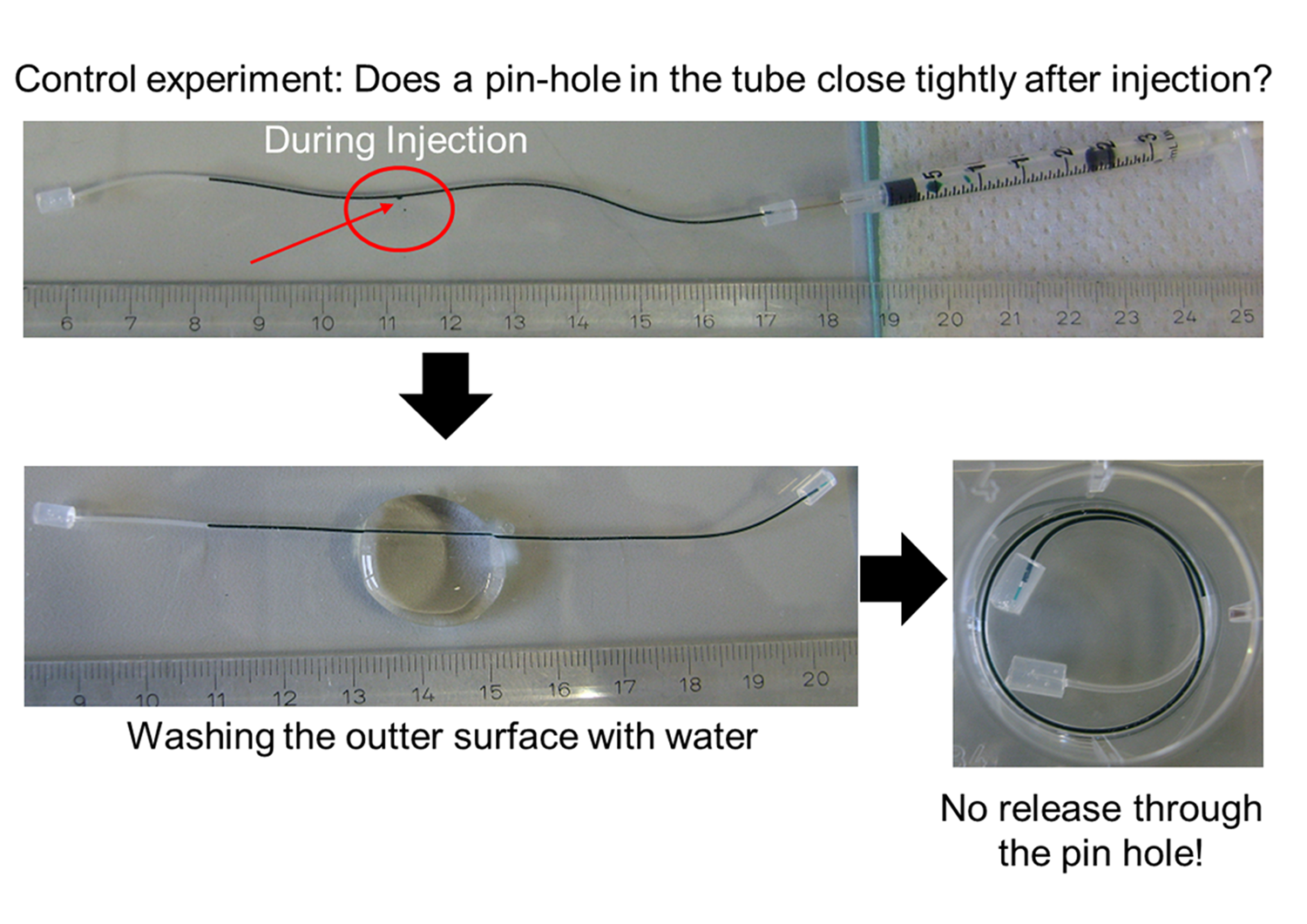

Supplement: Figure S5 — Control experiment using a dye-solution showing that the pin-hole created in the silicone tube during loading of the DEX solution does not result in uncontrolled release. (TIF) [file pone.0104564.s005.tif]

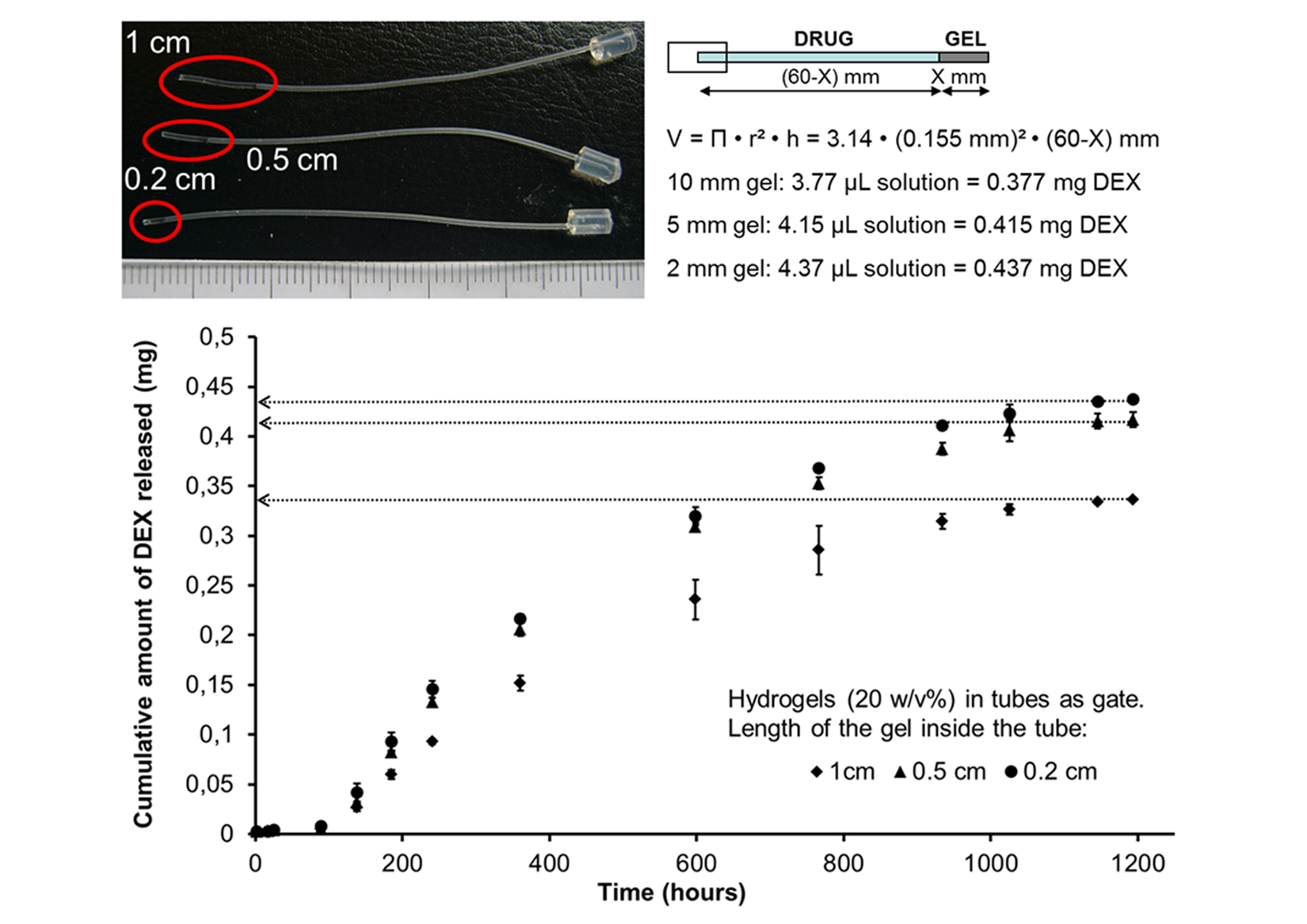

Supplement: Figure S6 — Picture of hydrogel-gates with three different lengths in silicone tubes (top left), calculation of the amount of DEX in each of the tubes (top right) and result of the release studies (bottom). (TIF) [file pone.0104564.s006.tif]

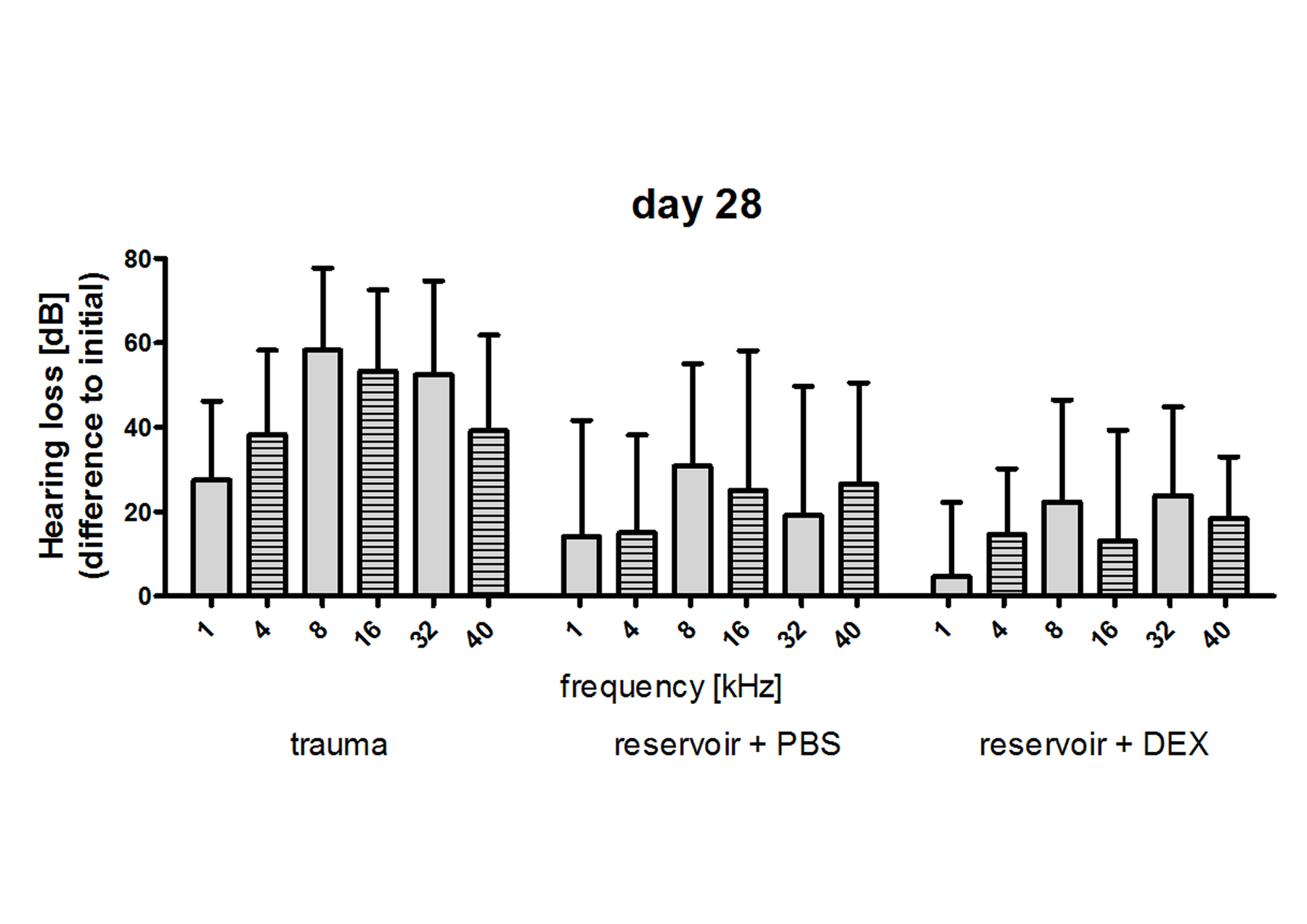

Supplement: Figure S7 — Frequency specific hearing loss. The mean and SD of hearing loss of all experimental groups after 28 days of implantation is plotted for each frequency tested. In all groups the hearing loss seems to be less affected in the lower frequencies but statistical evaluation did not show any significant differences between the frequency specific hearing loss in any of the experimental groups. (TIF) [file pone.0104564.s007.tif]

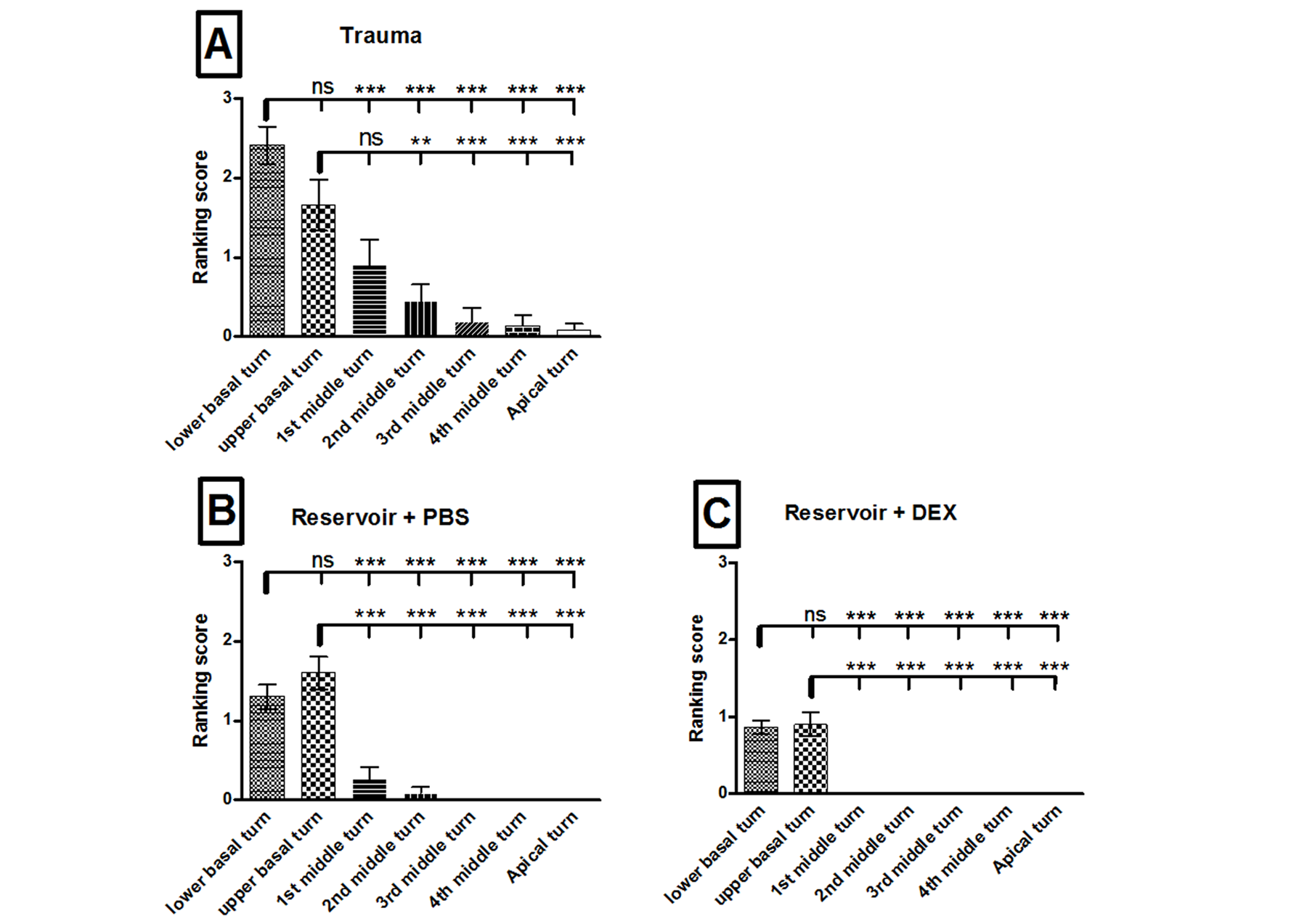

Supplement: Figure S8 — Tissue growth evaluated by ranking. The mean ± SEM results of subjective ranking of tissue formation in the cochlea turns of each experimental group are plotted. In all groups the tissue reaction is significantly increased in the basal regions compared to the middle and apical regions. Fibrotic tissue response in more apical turns was only detected in the trauma group (A) and did not take place in reservoir groups treated with PBS (B) or DEX (C). One-way ANOVA in combination with the Tukey post-test was used to compare the tissue growth within the different cochlea turns of each experimental group: ** = p<0.01; *** = p<0.001. Reference of the significance is marked by the thick bar. (TIF) [file pone.0104564.s008.tif]

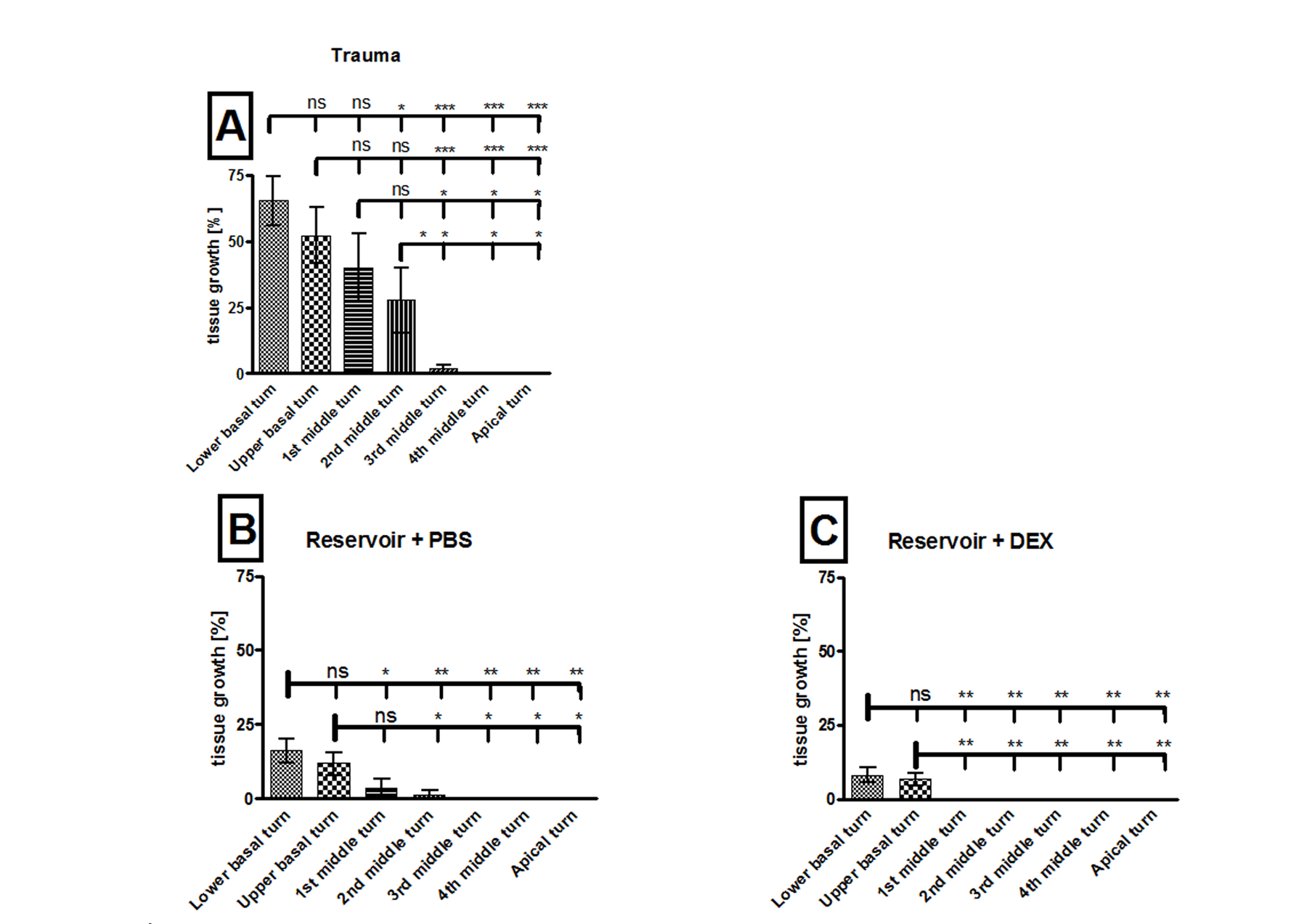

Supplement: Figure S9 — Tissue growth evaluated by measurement. The mean ± SEM percentage of scala tympani area of each cochlea turn covered with tissue is plotted for each experimental group. In all groups the tissue reaction is significantly increased in the basal regions compared to the middle and apical regions. Fibroblast growth in more apical turns was only detected in the trauma group (A) and did not take place in reservoir groups treated with PBS (B) or DEX (C). One-way ANOVA in combination with the Tukey post-test was used to compare the fibrous tissue growth within the different cochlea turns of each experimental group: ** = p<0.01; *** = p<0.001. Reference of the significance is marked by the thick bar. (TIF) [file pone.0104564.s009.tif]
